# Gender inclusivity through maize breeding in Africa: A review of the issues and options for future engagement

**DOI:** 10.1177/00307270211058208

**Published:** 2021-11-22

**Authors:** Rachel C. Voss, Jason Donovan, Pieter Rutsaert, Jill E. Cairns

**Affiliations:** 1 535603 International Maize and Wheat Improvement Centre (CIMMYT), Nairobi, Kenya; 2 535603 International Maize and Wheat Improvement Centre (CIMMYT), Texcoco, Mexico; 3 535603 International Maize and Wheat Improvement Centre (CIMMYT), Harare, MP, Zimbabwe

**Keywords:** Gender, maize, crop breeding, improved seeds, farmer preferences, Africa

## Abstract

With the prioritization of social inclusion in agricultural development, donors and research centers have shown growing interest in gender-intentional varietal development and delivery. Breeding maize varieties that respond to gender-based differences in trait preferences now represents a central objective of maize R&D in the CGIAR and elsewhere. Drawing on literature on gender and maize seed adoption, variety preferences, and seed system constraints, we take stock of knowns and unknowns related to gender-responsive and gender-intentional maize breeding. While recent research on farmers’ variety preferences across crops has yielded insights into gender-based differences, we find that evidence of gender-differentiated preferences for maize varieties remains inconclusive. Ultimately, we identify several research priorities to support gender-intentional maize breeding, including a more nuanced understanding of gender relations in maize production and maize seed decision-making, new and more gender-responsive approaches to measuring farmer preferences and seed demand more broadly, and research to address operational challenges in gender-intentional breeding. We close by identifying some institutional constraints to achieving impact through gender-intentional maize breeding.

## Introduction

Over the last fifty years, interest in gender relations and equity concerns have grown from a niche concern of feminist economics to a major focus of agriculture development (Cornwall and Rivas, 2015). Gender-related programming now features prominently in donors’ investment portfolios and development agencies’ strategies, providing an encouraging sign of their commitment to progress on equality and social inclusion. However, relatively limited evidence exists on how to effectively integrate gender-related concepts and approaches into diverse aspects of agricultural development. Such integration has been especially challenging given the complexities involved and the time, budget, and staffing constraints faced by development projects, as highlighted in a recent review by Stoian et al. (2018) on gendered programming for value chain development. Unsurprisingly, perhaps, strategies to mainstream gender in development have attracted criticism for failing to meaningfully incorporate feminist critiques or substantially change the way development agencies and initiatives function (Cornwall and Rivas, 2015; de Waal, 2006; Farhall and Rickards, 2021; Harcourt, 2016; Moser, 1989; Smyth, 2007). As such, critical reflection on past experiences and innovative approaches to gender integration in agricultural development are warranted.

Gender integration in crop breeding programs has attracted substantial interest and investment, as the development and dissemination of improved varieties forms a key component of development strategies aimed at smallholder production systems in sub-Saharan Africa and elsewhere. Donor-supported breeding efforts are largely centered at CGIAR crop research centers and their partners, which focus on breeding improved varieties of a diverse set of crops important for smallholders, including maize, wheat, rice, potatoes, cassava, sorghum, and bananas (CGIAR, 2018). These breeding programs are looking at how to advance an agenda around socially inclusive breeding with support from social science researchers (CGIAR Excellence in Breeding Initiative, 2020; Orr et al., 2018; Tufan et al., 2018). At a minimum, these breeding efforts aim for greater gender-responsiveness by understanding women's and men's interests, needs, and constraints; incorporating these into planning and decision-making related to breeding; and monitoring for any possible gendered impacts of new varieties to ensure they do no harm to women through, for example, increased labor requirements or reduced nutritional quality (Ashby and Polar, 2021). Some projects go further, pursuing gender-*intentional* breeding that targets women as an important but underserved market segment and aims to generate varieties with the potential to directly benefit women and reduce gender inequalities. Both gender-responsive and gender-intentional breeding approaches seek to reverse the historic exclusion of women's preferences and priorities from breeders’ understanding of smallholders’ needs, primarily by better assessing and responding to potential gender-based differences in crop requirements (Weltzien et al., 2019).

In this paper, we focus on gender and maize breeding efforts in sub-Saharan Africa, especially in eastern and southern regions. One of the most important cereal crops for smallholders in sub-Saharan Africa, maize covers almost 40 million hectares (FAO, 2021) and supplies a central part of both household diets and incomes. As such, maize is a major focus of breeding work in the CGIAR and national agricultural research centers, with outputs expected to play a central role in future food security in sub-Saharan Africa. Pursuit of gender and social inclusion objectives in maize breeding has led to a search for evidence of gender-differentiated maize preferences and market segments (MacNeil, 2020) as well as inclusive approaches to seed marketing and delivery (Bossuet, 2020). To the best of our knowledge, no paper has yet to take stock of our collective understanding of how gender influences maize seed adoption, what is known and unknown about gender and maize trait and variety preferences, and how research on gender and maize breeding should progress.

In the sections that follow, we explore the literature on maize seed adoption, evidence around gender-differentiated preferences for maize—whether research has identified traits preferred disproportionately and consistently by women maize farmers and women consumers, and the extent to which these preferences translate into variety uptake—and seed system constraints relevant to gender and maize work. We review some of the methodological and conceptual challenges to better understanding women and men's seed preferences and purchasing behaviors, outlining an agenda for future research on gender and maize. This agenda includes building a more nuanced understanding of gender relations in maize production and maize seed decision-making, exploring new and more gender-responsive approaches to measuring farmer preferences and seed *demand* more broadly, and working to address operational challenges in gender-intentional breeding, particularly by generating the insights required to inform trait prioritization and market segmentation.

## Gender and uptake of improved maize seed

A central justification for gender-responsive and gender-intentional breeding stems from disappointing adoption and turnover rates for modern, improved varieties (McEwan et al., 2021; Morris and Bellon, 2004; Spielman and Smale, 2017; Thiele et al., 2020). While decades of expert-led crop breeding have increased genetic gains for yield and produced hundreds of new varieties (Cobb et al., 2019; Evenson and Gollin, 2003; Walker and Alwang, 2015), farmer uptake of modern varieties has been mixed. Adoption of improved varieties of ten key food crops in Africa ranged between only 31% (pearl millet) and 64% (wheat), with modern maize varieties falling in the middle at 52% (Walker et al., 2015). While these figures mask substantial spatial variation in uptake, they do suggest generally modest rates of adoption of improved varieties. Slow adoption of new varieties has frustrated many, not only for the resulting limited returns on investments in breeding, but for the failure to generate intended benefits for smallholders.

Furthermore, there is evidence that uptake of improved varieties is unequal between men and women, drawing attention to gender issues in breeding and seed systems. Maize is one crop that shows evidence of a gender gap in adoption of improved varieties, although it manifests differently across countries and studies. In Uganda, women-headed households were found to be 26% less likely to use drought tolerant (DT) maize than men-headed households, and wives in spousal-couple households to be 16% less likely (Fisher and Carr, 2015). Another study from Uganda found that awareness of DT maize was 67% for male household heads, 51% for wives, and 43% for female household heads, while adoption rates followed similar patterns: 29% for male household heads, 11% for their wives, and 5% of female household heads (Fisher et al., 2019). Both female household heads and women in male-headed households in Malawi were less likely to grow modern maize, although subsidies appeared to boost adoption over 200% among female-headed households (Fisher and Kandiwa, 2014). In Ethiopia, one study found that improved maize use in female-headed households was 29% lower than in male-headed households (Kassa, 2013). However, a more recent study found that the rate of use of improved maize varieties did not differ according to the gender of the decision-maker for maize plots, but the *intensity* of production did vary, and rates of improved maize adoption were positively correlated with the number of adult males in the household (Gebre et al., 2019). In Kenya, similarly, intensity of use of hybrid seed was lower among female-headed households, which used, on average, 1.8 kg less hybrid seed than male-headed households (Smale and Olwande, 2014). Recent work in Zimbabwe documented differences in variety use among male and female plot managers (Cairns et al., 2021b), but clear explanations of these differences are still needed.

Most of this research has gone beyond gender-based comparisons in search of explanations of adoption patterns, often regressing seed use or *intensity* of seed use on household and individual farmer characteristics. Studies indicate that the factors most commonly associated with adoption of new agricultural technologies include access to labor, land, cash and credit for inputs, extension, education, and decision-making authority—access to all of which is mediated by social norms that dictate roles and responsibilities for women in the household and in society (Doss, 2001; Quisumbing and Pandolfelli, 2010). As such, adoption research has frequently found that gender becomes an insignificant predictor of improved seed uptake when other farmer characteristics that correlate with gender, particularly access to complementary inputs, are controlled for (Bezu et al., 2014; Doss and Morris, 2001; Fisher et al., 2019; Ndiritu et al., 2014; Peterman et al., 2014).

Notably, farmer trait preferences are rarely included as possible predictor variables in maize adoption studies. Exceptions from sub-Saharan Africa include Lunduka et al.'s (2012) analysis of farmers’ use of modern, open-pollinated, and local varieties of maize in relation to the traits farmers identify as important, which underscored that farmer valuation of traits correlates with seed choices. Fisher et al.'s (2015) six-country study of DT maize adoption also incorporated trait preferences, finding that farmers’ valuation of yield, drought tolerance, and early maturity were among the variables correlated with adoption of DT varieties. While gender was not a focus of either study, these results suggest a potentially important knowledge gap in the literature on gender and improved maize adoption: the role of preferences in guiding men's, women's, and joint seed choices. If women's unique preferences for seed substantially influence their seed uptake decisions, identifying and integrating their preferences through gender-responsive and gender-intentional breeding efforts is key.

## Gender-differentiated variety preferences

The concern that women's uptake of improved varieties lags behind men's due to the differentiated acceptability of modern varieties is one driver of CGIAR crop research centers’ focus on more gender-responsive and gender-intentional product profile design. This emphasis is largely donor-driven; gender considerations now feature prominently among the priorities of crop breeding funders and breeding training programs (Bill & Melinda Gates Foundation, 2021; CGIAR Excellence in Breeding Initiative, 2020; GREAT, 2021; Orr et al., 2018). Gender-responsive breeding also constitutes a response to broader criticisms that CGIAR breeding programs have historically operated with limited farmer participation, resulting in inattention to performance under diverse agroecological conditions and to some farmer-preferred traits (Ashby and Polar, 2019; Forsythe et al., 2020; Haugerud and Collinson, 1990; McEwan et al., 2021; Morris and Bellon, 2004; Thiele et al., 2020).

The CGIAR Gender and Breeding Initiative (GBI), launched in 2017, has published tools and piloted approaches for breeders and social scientists to better capture and act upon farmers’ needs and priorities (Ashby and Polar, 2021; CGIAR Excellence in Breeding Initiative, 2020; Mudege et al., 2020; Orr et al., 2018; Ragot et al., 2018). It guides gendered analysis of market segments, supports identification of gender-relevant trait(s) and their addition to the set of formal criteria used to develop and evaluate new varieties, and presents a roadmap for making gender-intentional breeding decisions (Ragot et al., 2018). The design and piloting of GBI approaches has been based largely on experiences in a specific set of crops, particularly cassava, potatoes, bananas, and sweet potatoes (Forsythe et al., 2020; Ndjouenkeu et al., 2021; Teeken et al., 2020; Thiele et al., 2020). These tools will need to be further validated through use on cereals and other crops where end-use traits may be less important in variety uptake decisions.

Logically, a central focus of GBI guidance is on identifying the traits and varieties that women prefer disproportionately relative to men and vice versa. Recent reviews of gendered trait and varietal preferences across a range of crops have suggested traits preferred disproportionately by women and men do exist (Christinck et al., 2017; Weltzien et al., 2019). Most notably, women often prioritize end-use traits such as shellability, milling characteristics, water absorption, speed of cooking, taste, and storability, likely due to their role in cooking, processing, and storage activities (Weltzien et al., 2019). Reviews have also suggested that women disproportionately value agronomic traits that impact household food security—for instance, early maturity (which cuts short the lean season and mitigates the risk of late-season drought in regions with unimodal rainfall patterns) and consistent production under less-than-ideal conditions. Men, in contrast, tend to prioritize a narrower range of traits related to production and marketability (Weltzien et al., 2019). These patterns, while not necessarily universal across farmers, locations, or crops, do highlight possible areas of interest for research on maize preferences.

### Gender and maize variety preferences

From a gender and breeding perspective, maize is unique in a few important ways, necessitating thoughtful approaches to preference research. Maize is consumed in several forms across sub-Saharan Africa—boiled or roasted fresh; shelled, dried, and rehydrated as whole grain for incorporation into dishes; wet-ground and steamed; dry-ground and used to create breads, dumplings, or porridges; or distilled into alcohol (Ekpa et al., 2018, 2019). However, the range of end-uses are often relatively narrow within a specific context. As a commodity crop consumed largely in flour form, variation in maize end-use characteristics is arguably not as important or desirable as it might be for roots, tubers, and legumes, which are principally consumed by the household and are frequently distinguished by their suitability for specific foods. As such, donor investments in CGIAR and national maize breeding programs have prioritized production traits rather than consumption traits in the past, producing higher yielding and more stress-tolerant varieties (Cairns et al., 2021a; Rezende et al., 2020; Setimela et al., 2017; Worku et al., 2020). These varieties are then produced and delivered primarily by privately owned seed businesses and retailers.

While production of some crops is gendered, leading to distinct and relevant gender-based differences in trait preferences, maize production is *not* distinctly divided along gender lines in many contexts. Studies have shown that women are generally more involved than men in production of roots, tubers, bananas, beans, and vegetables for household consumption, with men more likely to produce cash crops (Assefa et al., 2014; de Brauw, 2015; Grassi et al., 2015; Sperling et al., 1993; Weltzien et al., 2019)—although many of these distinctions are less clear (Doss, 2002; Orr et al., 2016) and more mutable (Geisler, 1993) than they often appear. For crops grown disproportionately by women for household consumption, gender-intentional breeding frequently focuses on end-use traits. Maize, however, is important for both household food security and income generation. It rarely falls cleanly into either men's camp or women's and tends to be grown widely by both men- and women-headed households (e.g. Cairns et al., 2021b; Doss, 2002). In East Africa, research has shown that responsibilities for its production are often shared between men and women, either in separate or jointly managed plots (Adam et al., 2020a; Adam et al., 2020b). There is evidence of gendered divisions of production activities when maize is jointly produced, but these vary by context. The clearest distinctions emerge in relation to weeding, processing, and cooking, which are almost exclusively the domain of women, as they are for many other crops (Badstue et al., 2020; Doss, 2001; Gouse et al., 2016). However, because maize production is multi-purpose and often a shared household responsibility, gender-intentional maize breeding must account for gender-based differences in preferences surrounding both end-use characteristics and agronomic traits.

The imperative to incorporate farmer preferences into maize breeding processes has long existed. Maize breeding programs in sub-Saharan Africa have focused on several key agronomic traits valued by smallholders, including performance under climate change, in less-than-ideal growing conditions, and in diverse agroecological contexts (Cairns et al., 2021a; Cairns and Prasanna, 2018). Most of the preference data supporting this work emerged from on-farm variety evaluations, described in detail below. Breeding efforts have also touched on end-use traits, most notably on color preferences (Ekpa et al., 2018) and grain texture. In the 1980s-90s, researchers documented smallholders’, and especially women's, preference for flint varieties, which are more easily converted to flour and typically store better than dent varieties (Mulatu and Zelleke, 2002; Smale and Heisey, 1994). Although maize mills are increasingly common in some places, pounding maize was, at that time, a manual task performed by women in many places, including Malawi. The identification of and breeding for this gender-relevant trait in Malawi led to much wider production and use of flint varieties and modern varieties generally (Lunduka et al., 2012). Still, slow varietal turnover and farmers’ indifference toward new seed options (Rutsaert and Donovan, 2020; Smale and Olwande, 2014; Spielman and Smale, 2017) provide circumstantial evidence that maize breeding programs may not yet appeal optimally to farmers’ production- and consumption-related priorities, needs, and constraints.

Over the last two decades, maize researchers have further examined farmers’ stated and revealed seed preferences through participatory varietal selection (PVS), choice experiments, experimental auctions, and surveys. Several studies using diverse methods have found no conclusive differences between men and women's preferences for maize. De Groote et al. (2002) used open-ended gender-disaggregated focus groups to discuss and rank important maize traits and constraints, then conducted on-station trial evaluations of agronomic properties up to the point of harvest, but they found no significant differences between women and men's trait selections. Similarly, using household surveys and choice experiments in Uganda, Fisher and Carr (2015) found no significant differences in men and women's perceptions of drought risk or their preferences for yield or drought tolerance. In Zimbabwe, despite some variation in women's and men's variety preferences, their valuation of agronomic traits did not reveal clear differences (Setimela et al., 2017).

Even where studies detected variation in preferences, differences were often minor and showed few consistent patterns. Ultimately, yield, early maturity, and stress tolerance typically emerged as top traits for maize regardless of gender. For example, a multi-country study of gender and maize variety preferences drawn from PVS in West Africa found that while drought tolerance, yield, maturity period, and several other characteristics were valued by both men and women, men showed somewhat greater focus on marketability and women on grain color, appearance, and food quality (Tegbaru et al., 2020). Recent work by Worku et al. (2020) based on preference evaluations in on-farm maize trials in Kenya and Rwanda found that women and men's stated evaluation criteria were largely the same. However, in the variety evaluations, women were slightly more likely to prioritize yield, early maturity, and cob size, while men valued stalk thickness, foliar disease resistance, barrenness level, and stalk borer resistance, among other factors. Still, these differences were not substantial enough to impact top trait priorities.

Women's disproportionate interest in end-use traits appears in many maize studies in Africa, including in Benin (Baco et al., 2015), Ethiopia (Mulatu and Zelleke, 2002), Mali (Defoer et al., 1997), Mozambique (Adam et al., 2020b), Uganda (Fisher and Carr, 2015), and multiple West African countries (Tegbaru et al., 2020). A few studies have also indicated that men's maize trait preferences skew toward production and marketability—for instance, yield and grain size—while women value traits lending them more stable production under variable and unpredictable conditions (Adam et al., 2020b; Mulatu and Zelleke, 2002; Setimela et al., 2017) or reduced production labor (Gouse et al., 2016). However, these traits often emerge alongside or secondary to yield and stress tolerance traits, making it difficult to infer the importance of these gender-differentiated trait preferences in variety choice.

A few studies have used choice experiments to deepen our understanding of farmer decision-making, particularly around trait trade-offs. Kassie et al. (2017) examined differences in preference between male- and female-headed households in Zimbabwe, where male household heads expressed more interest in yield and semi-flint texture, while female household heads were more willing to pay for large cobs (which can be easier to shell and sell well as fresh maize). However, any analysis by gender of household head introduces important confounding variables, as discussed later. Marenya et al. (2021) conducted a choice experiment and experimental auction study in Kenya involving *individual* men and women farmers (including women in male-headed households), finding similar trait preferences among men and women but differences in willingness to sacrifice yield in exchange for other traits. In this study, women were 2–3 times more willing than men to sacrifice more yield in exchange for greater storability, drought tolerance, and *Striga* resistance, while men were more likely to sacrifice yield in exchange for good ear tip cover (Marenya et al., 2021). Althought these studies rely on hypothetical decision-making, they move us further toward understanding if, when, and how gender-relevant traits should figure into breeding decisions.

Gender-based trait and variety preference data theoretically provide valuable insight into the acceptability of improved maize varieties for women and men—a prerequisite to adoption. However, taken together, the existing research on gender and maize preferences provides little clear evidence of gender-differentiated maize trait preferences that contribute to discrepancies in improved seed adoption. Studies have leveraged diverse methods in varied contexts and, unsurprisingly, generated inconsistent results. Consequently, breeding programs have a weak evidence base for shifting investments from one product profile to another or for creating new, more gender-responsive product profiles. Still, methodological challenges and inconsistencies, discussed later, suggest that the question of gender-based differences in maize preferences may not be fully answered. Furthermore, preference research only addresses one dimension of the adoption challenge. Substantial questions remain beyond the scope of maize preference studies, including the business case for gender-relevant traits’ incorporation into new varieties and gender inclusivity in maize seed systems, broadly.

## Gender-related constraints in maize seed systems

The focus on identifying gender-relevant traits—which are the key input breeders need to inform product design—assumes problems about the appeal and acceptability of existing maize varieties to women. However, preference studies do not evaluate the extent to which better integrating women's preferences into breeding efforts would increase demand for new maize varieties, drive higher adoption rates among women, or contribute to gender equality. This is particularly uncertain given clear evidence that the vast majority of maize farmers appear uninterested in trying new varieties (Rutsaert et al., 2021). Undoubtedly, breeding efforts that broaden the appeal of improved maize must be paired with seed system interventions to increase the availability of, farmer interest in, and equal access to new gender-intentional offerings. This includes better understanding and addressing seed availability and accessibility, the gender dynamics of information access, and intrahousehold decision-making around seed.

Expanding the availability of existing or new maize seed offerings is likely to be more challenging than for other cereal crops. The uniquely high degree of dependence on the private sector for maize seed delivery means that the release, production, marketing, and distribution of improved maize seeds are rarely pro-poor or gender-responsive (Access to Seeds Foundation, 2019; Brearley and Kramer, 2020). Research across crops has generated suggestions for more gender-responsive seed marketing and distribution systems (Adam et al., 2019; Audi et al., 2015; Kandiwa et al., 2018; Louwaars et al., 2013; Sperling et al., 2021; Sperling and Boettiger, 2013). However, most of these approaches have yet to be rigorously evaluated, so the business case for private sector investment in these areas is not yet clear. Subsidies or other incentive structures to support equitable maize seed access may be needed.

Seed accessibility is another substantial challenge. As reviewed above, decades of seed adoption research have shown that resource access often plays a significant role in predicting adoption behavior, while gender itself (and any preferences inherently associated with gender) frequently does not (Bezu et al., 2014; Doss, 2001; Fisher et al., 2019; Ndiritu et al., 2014; Peterman et al., 2014). Resource access includes information access, which is frequently skewed in terms of gender. Women are frequently more likely to rely on family and neighbors for farming information and less likely to engage with public extension services (Brearley and Kramer, 2020; Meinzen-Dick et al., 2011; Quisumbing and Pandolfelli, 2010; Ragasa, 2014). The role of information and seed accessibility in constraining use of improved maize varieties has been examined, but primarily at the household level (e.g. Simtowe et al., 2019) rather than among individuals.

Existing literature provides insight not only on the importance of seed availability and resource access in expanded seed access, but increasingly on the importance of women's agency. The GENNOVATE initiative highlighted how social norms at the household, community, and society level undermined women's ability to choose, purchase, and productively use new technologies (Badstue et al., 2020; Farnworth et al., 2020; Petesch et al., 2017, 2018). Indeed, studies from sub-Saharan Africa have underscored that women's involvement in maize seed decisions are often superficial (Acosta et al., 2020; O’Brien et al., 2016). This means, for example, that evidence of women's relative willingness to sacrifice yield in exchange for other traits (Marenya et al., 2021) may not ultimately influence household seed decisions. Addressing gender inequality through breeding will require simultaneous attention to women's preferences and to their ability to choose, acquire, and use improved seed—and therefore their agency within households.

## An agenda for future research in gender and maize breeding

The gender and maize research reviewed above draws on diverse methodological approaches to understanding gender-based variation in maize preferences and seed use. Ultimately, however, it provides little conclusive guidance for gender-intentional maize breeding. More studies in the same vein may or may not provide clearer answers. Critical reflection, innovative methods, and more coordinated studies are likely needed, as well as consideration of alternative impact pathways. Here, we outline an agenda for the next generation of research on gender and maize breeding that includes 1) developing a more nuanced conceptual understanding of maize production, seed choice, and seed acquisition dynamics, 2) implementing innovative approaches for understanding preferences, and 3) addressing operational challenges to gender responsive breeding.

### Toward a more nuanced understanding of gender and maize systems

There is much evidence to suggest that the real-world gender dynamics of maize production, seed choice, and seed acquisition are more complicated than existing gender and maize research assumes. An implicit assumption underlying much research on gender and agriculture concerns the independence of farm management and decision-making among men and women. Studies comparing maize seed choice between individual men and women or between men's and women's plots (Fisher and Carr, 2015; Marenya et al., 2021; Ndiritu et al., 2014; O’Brien et al., 2016) struggle to accommodate scenarios wherein maize farming is practiced jointly under complex decision-making arrangements (Acosta et al., 2020; Anderson et al., 2017; Doss and Quisumbing, 2018; Theis et al., 2017). How management decisions are made on ‘women's plots’ and ‘men's plots’ is rarely explained, so the implications for seed decision-making are unclear. By extension, treating farmers’ individual preferences as predictors of maize seed decisions is problematic. This conceptual challenge aligns with growing recognition that concepts such as ‘women's crops’ or ‘women's productivity’ tend to mask substantial diversity, dynamism, and complex intrahousehold relationships (Doss et al., 2018; Doss and Quisumbing, 2020; Orr et al., 2016). Furthermore, spatial variation in gender roles in maize management make the challenge of interpreting preferences and seed choice even more complex for breeders.

Some econometric studies of preferences and seed adoption rely on comparisons between female-headed and male-headed households as a means toward gender disaggregation (e.g. Kassie et al., 2017; Simtowe et al., 2019). This avoids some of the complexity of plot management dynamics, but household headship is a deeply problematic proxy for gender due to the structural differences and resource access gaps between these household types (Doss, 2015). Furthermore, subjective and varied interpretations of what household headship entails (Budlender, 2003; Posel, 2001; Rogan, 2016) undermine household comparisons as a means to understand gender dynamics. Neither of these approaches—reliance on comparison of men's and women's plots or male- and female-headed households—are grounded in an adequate understanding of gender relations in maize production.

More research is needed to understand how norms and intrahousehold dynamics shape women's roles in maize production broadly and maize seed choice in particular. The toolkits developed through GENNOVATE provide models for capturing women's lived experiences and barriers to experimentation with new maize varieties (Badstue et al., 2018; Petesch and Bullock, 2018). However, because an understanding of broader patterns is needed to inform breeding efforts, qualitative approaches to understanding maize production dynamics should be paired with innovative quantitative tools for capturing preferences across broader regions.

### Generating better maize preference data

Tied to the need for a deeper understanding of intrahousehold dynamics and joint plot management is the need for new methodological and conceptual approaches to understanding farmer preferences. The maize preference evaluations highlighted above leveraged different units of analysis (men vs. women farmers or plot holders, women- vs. men-headed households) and a range of approaches (stated preferences, variety evaluations, and revealed preference studies). Methodological inconsistency has likely contributed to the lack of clarity around gender-differentiated preferences—as have problematic assumptions underlying popular approaches.

#### Limitations of participatory varietal selection in understanding maize preferences

One of the most common tools for maize preference evaluation in recent years has been participatory varietal selection (PVS). PVS approaches vary, but in many maize preference studies, evaluations by men and women farmers were built into trials designed for varietal testing and comparison. The trials are either researcher-managed or farmer-managed to varying degrees (Schmidt et al., 2018) and can involve dozens of varieties grown under high- or low-input conditions in the same plot (De Groote et al., 2002; Setimela et al., 2017). Men and women farmers in the surrounding area are sometimes invited to participate in the trial, but at other times simply tour the trial and complete a survey evaluating the varieties planted—often but not exclusively at harvest (Misiko, 2013; Worku et al., 2020). This approach streamlines the process of collecting important farmer feedback for centralized breeding programs at minimal added cost, as field trials are used for both variety testing and PVS.

However, PVS can have important limitations and be gender-blind, depending on how it is implemented (Misiko, 2013). First, because trials are often wholly or partially ‘researcher-managed,’ they tend to be higher yielding than both national averages and participating farmers’ own fields (De Roo et al., 2017; Laajaj et al., 2020). CGIAR maize breeding programs led by the International Maize and Wheat Improvement Center (CIMMYT) tend to conduct on-farm varietal trials that are similar to experiment station trials, with researchers supplying inputs and excluding farmer practices such as intercropping that could reduce statistical power to interpret and compare trial results (Setimela et al., 2017; Worku et al., 2020). Given evidence that women practice intercropping more than men (Ndiritu et al., 2014; Tufa et al., 2019), these PVS design choices could have substantial implications for understanding gender-differentiated preferences.

PVS approaches can also inherently limit which traits farmers are able to observe and evaluate. As germplasm move through the breeding pipeline, genetic variability narrows. On-farm trials where PVS is frequently conducted represent the final stage of the pipeline at research institutes like CIMMYT, so genetic variability is limited; if variation for a trait is not present within trials, farmers cannot observe and evaluate it. Moreover, researchers determine which traits farmers are invited to observe and evaluate in trials—sometimes this process involves farmer input, but not consistently. Researchers can embrace more open-ended evaluation (e.g. Defoer et al., 1997; Mulatu and Zelleke, 2002) rather than asking farmers to quantify the importance of a prescribed list of traits (e.g. Baco et al., 2015; Worku et al., 2020). The former approach draws on experiences from participatory plant breeding and has the potential to better capture farmer priorities, including gender-specific preferences that deviate from outcomes that researchers expect.

Researchers can also unintentionally limit evaluation criteria via the choice of PVS timing. Most farmer variety evaluations of maize at CIMMYT (e.g. Setimela et al., 2017; Worku et al., 2020) are conducted at very specific maturity stages, typically immediately pre- or post-harvest, which narrows the range of criteria on which varieties are assessed to agronomic properties observable at those stages. Thus, even when these evaluations involve women, they are not usually structured to investigate gender-relevant properties related to processing, cooking, post-harvest storage, or performance under less ideal conditions. This can lead to “impulse buying,” whereby farmers select for conspicuous traits they *like* that do not necessarily reflect what they *need* (Misiko, 2013). Research that encompasses post-harvest evaluations are rare but do exist (examples for maize include De Groote et al., 2014; Defoer et al., 1997; Mulatu and Zelleke, 2002). In some studies, men and women evaluated maize at different stages; for example, in Mulatu and Zelleke (2002), only men were interviewed at full vegetative growth while only women were interviewed for flour preparation, which disregarded women's interest in agronomic performance as well as men's interest in consumption-related traits.

#### New approaches to understanding maize preferences

Better understanding gender-based differences in maize preferences will require new approaches at multiple levels, including the use of larger samples across wider geographic scales, more gender-responsive approaches, and more consistency and coordination across sites. GBI's pioneering work provides guidance to standardize preference studies and their interpretation across countries and crops and should be leveraged (Ashby and Polar, 2021; Ragot et al., 2018). However, innovative methods to collect farmer preference data at scale and in the context of their household production and livelihood systems are also needed. These have the potential not only to uncover gender-based differences in preferences, but to identify maize market segments more generally, including those disproportionately occupied by women.

Farmer-managed varietal trials across large geographic areas are one promising option. For example, triadic comparisons of technologies (tricots) offer means to involve a wide diversity of farmers in simple variety evaluations within their household context (van Etten et al., 2019a; van Etten et al., 2019b). Internet-based tools for choice experiments such as ‘1000 Minds’ have been used in animal breeding programs to capture farmer trait preferences across broad geographic areas and could be applicable here (Byrne et al., 2012; Martin-Collado et al., 2015). However, donor mandates to quantify new varieties’ genetic gain in yield and additional costs associated with decentralized methods mean such approaches will not easily replace researcher-managed trials. More work is needed to tailor these tools to generate key insights needed for maize breeding programs and seed systems interventions.

Finally, it is worth drawing lessons from older but effective approaches to incorporating farmer preferences into breeding. In the 1980s, development practitioners’ and researchers’ growing interest in participatory research (Chambers, 1994) led to the rise of participatory technology R&D, notably participatory plant breeding (Ashby and Lilja, 2004; Ashby and Sperling, 1995; Ceccarelli and Grando, 2019; Tufan et al., 2018; Witcombe et al., 2005). Participatory plant breeding (PPB) engages farmers at earlier stages in the product development process than PVS, including pre-breeding priority setting and germplasm selection rather than only post-breeding varietal testing and selection. Various case studies documented how PPB led to products that were more appealing to farmers and more readily adopted (Ashby and Lilja, 2004; Witcombe and Yadavendra, 2014). While PPB was not developed with gender inclusivity in mind, projects that increased involvement of women in trait identification and priority setting saw greater product uptake by women (Ceccarelli and Grando, 2007; Farnworth and Jiggins, 2003; Tufan et al., 2018) as well as women's empowerment (Galiè, 2013; Galiè et al., 2017). PPB is unlikely to be fully compatible with centralized maize breeding programs, but these efforts highlight the value of engaging farmer as collaborators in the breeding process rather than only end-users, and exploring opportunities for them to direct the breeding agenda. De Sousa et al.'s (2021) study of data-driven decentralized wheat breeding, for example, generated results robust enough to support moving earlier stages of breeding programs on-farm.

#### Distinguishing between trait preferences and seed demand in maize

Taking steps to generate better and more gender-responsive data around individual maize preferences is important, but researchers and breeding programs must better understand how these preferences relate to seed demand. Future research to support gender-intentional maize breeding will need to expand conceptually to encompass maize seed demand and market segmentation, including how individual farmers’, households’, agroindustries’, and consumers’ needs, aspirations, and constraints influence seed decisions. This conceptual expansion will require new and innovative methods.

Many research methods currently employed to understand farmer preferences and decision-making bear little relation to farmers’ real-world seed acquisition (Almekinders et al., 2019) and poorly capture farmer *needs* (as opposed to *likes*) (Misiko, 2013). This may be particularly true for women, whose stated preferences in on-farm evaluations or choice experiments do not indicate a need or an ability to purchase preferred seeds given complex factors in and barriers to real-world adoption (Doss and Morris, 2001). An illustrative recent study of maize farmers in Zimbabwe (Cairns et al., 2021b) showed significant gender-based differences in varieties grown, but trait preferences identified in participatory varietal evaluations did not correlate with variety use; women liked the popular commercial maize hybrid Pan53 in researcher-managed on-farm trials, yet few women plot managers seemingly purchased this variety. The immediate reasons for this discrepancy are unclear, but it highlights the danger in relying heavily on preference studies to understand seed demand.

Choice experiments have contributed to an increased understanding of farmers’ willingness to make trait trade-offs in maize (Kassie et al., 2017; Marenya et al., 2021) and other crops (Demont and Ndour, 2015). These provide some insight into trait prioritization. However, the hypothetical nature of seed choice experiments, which do not allow farmers to experientially evaluate seeds, means their relevance to real-world seed choice is limited. Supplementing this research through field experiments and observational studies around seed choice could help validate insights from choice experiments.

More qualitative and dialogue-based approaches are also needed to enable inductive exploration of farmer needs and constraints in the specific agroecological, livelihood, and household context in which they select and purchase seeds (Almekinders et al., 2019; McEwan et al., 2021). This should include increased attention to intrahousehold decision-making dynamics (Acosta et al., 2020; Anderson et al., 2017; Doss and Quisumbing, 2020; van Campenhout et al., 2021), with the assumption that seed decisions do not happen in a vacuum. The GENNOVATE Initiative laid the groundwork for increased use of qualitative methods at scale, but pairing these tools with innovative quantitative analysis of maize preferences and needs is likely to be a critical step toward understanding seed demand at the scale required for breeding.

### Challenges in operationalizing gender-intentional breeding for maize

The final agenda item for future gender and maize breeding research relates to operationalizing gender-intentional maize breeding. One set of challenges is conceptual. First, gender-relevant traits must compete with many other breeding priorities, but there is little guidance in place for making decisions around the trade-offs involved. While in theory plant breeders can include an extensive list of traits in their selection criteria, the more traits included in selection, the lower the genetic gain for each trait. Individual countries impose specific requirements that varieties must meet in value for cultivation and use (VCU) testing. On average, 10 to 15 agronomic traits are included in the VCU testing, with up to 36 traits in Ghana (Setimela et al., 2009), making it difficult to elevate additional gender-relevant traits. Changing climatic conditions demand prioritization of yet more traits, particularly tolerance to biotic and abiotic stresses (Cairns and Prasanna, 2018; Prasanna et al., 2021). For this reason, maize breeding in sub-Saharan Africa has justifiably focused on yield, stress tolerance, and defensive traits to respond to emerging pest and disease threats, all of which will continue to be high priorities. Trade-offs will be required as gender-relevant traits replace or reduce the weight of other important variety characteristics in breeding pipelines.

Furthermore, at present, maize seed market segmentation is relatively simple, with farmers grouped according to their mega-environments. The clearer identification of farmer, agroindustry, and consumer preferences, including gender-relevant traits, would allow for more nuanced segmentation of the maize seed market—even more so if large-scale studies across sites uncover substantial variation in needs and constraints. In theory, current product profiles can be subdivided along gender and other socioeconomic lines, but this has implications for selection gains (Atlin et al., 2000) and cost-benefit ratios. Donors and breeders will need to acknowledge and weigh the inevitable costs that could result from breeding for more niche markets, as well as the lack of incentives for small- and medium-scale seed enterprises to embrace production and distribution of more tailored seed options.

Informed decisions around breeding trade-offs—for instance, how to balance gender-relevant traits with other breeding priorities and how much genetic gain should be sacrificed for finer-grained market segments—will require additional multidisciplinary research to evaluate costs and benefits. This work will need to dedicate specific attention to downstream benefits to women and households. It should also include analysis of alternative, non-breeding interventions to address needs; breeding is clearly not the only way to address gender concerns and may not be most effective since genetic improvements are inherently limited by genetic variation for relevant traits within the primary gene pool. Many maize storage and processing challenges could potentially be addressed more quickly, and likely more economically, through women's increased access to non-seed technologies.

Other challenges in operationalizing gender-intentional maize breeding are logistical in nature. For instance, the incorporation of any trait into a product profile requires major resource expenditures for development of phenotypic assays (where needed) and subsequent breeding efforts. This process is more complex in the case of gender-intentional breeding due to ambiguous or unquantifiable outputs from gender-disaggregated preferences evaluations. For example, in Benin, women identified appearance and organoleptic quality as important traits for maize, but the term ‘organoleptic’ refers to a multitude of traits such as aroma, appearance, taste, texture on the hand, or texture in the mouth (Baco et al., 2015; Tegbaru et al., 2020). Similarly, ‘grain quality’ (Setimela et al., 2017) pertains to numerous traits that may vary across and within geographies depending on end-uses. These traits must be translated into quantifiable, ‘breedable’ targets to enable design of phenotypic assays and incorporation into breeding programs, beginning with grain quality analysis paired with trait evaluation. Laying this groundwork for gender-intentional maize breeding, while complicated, is a clear next step for researchers.

## Conclusions and recommendations

In search of an agenda for the future of gender-intentional maize breeding, we combined insights from literature on improved maize seed adoption, trait and variety preferences, and maize seed systems. Adoption research has pointed to a range of factors influencing seed use, including access to resources and information and, potentially, farmer preferences and seed acceptability. Trait preference studies for diverse crops have yielded some valuable insights—notably women's heightened attention to end-use-related traits and agronomic characteristics that stabilize household food security. However, maize research has provided little clear evidence of gender-based differences in preferences that could direct breeding endeavors, in part because studies are fragmented and methodologically inconsistent.

In examining this research, we identified knowledge gaps and methodological issues that inform an agenda for future work around gender and maize breeding. Future research priorities include, first, stepping back to build a more nuanced understanding of gender relations in maize production and maize seed decision-making. Thus far, the focus of gender-intentional maize breeding research has been primarily on gender-based differences in preferences, as this is the key input breeders need to inform product design. However, it is problematic to assume that farmers’ individual trait and variety evaluations predict maize seed purchase and use, as seed choice may be heavily influenced by household dynamics. Furthermore, the absence of clear gender-based differences in maize preferences suggests that farmer gender is likely insufficient to explain seed choice. More attention is needed to maize seed market segmentation, encompassing farmers’, agroindustries’, and consumers’ needs, constraints, and priorities, with consideration of women's representation in different market segments. Second, we emphasize the need to explore new and more gender-responsive approaches to measuring farmer preferences and to expand research on seed *demand* more broadly; variety choice may be such a foregone conclusion for most farmers that breeding and marketing seed in relation to trait preferences is ineffective. Finally, we identify research needed to resolve operational challenges in gender-intentional breeding, particularly by generating the insights around trait prioritization, market segmentation, and breeding for end-use traits.

This is also a critical time to reflect on the relevance and importance of gender-intentional breeding in efforts to advance gender equality in agriculture. There are significant institutional barriers to realizing gains from gender-intentional breeding, beginning with incentive structures. To satisfy donor requirements around gender, breeding programs are asking gender researchers and social scientists to identify traits preferred disproportionately by women. However, success in breeding programs is often linked to the number of varieties picked up and multiplied by seed companies. Seed companies are fundamentally profit-driven, and their priorities may not align with gender equality goals. As such, existing incentive structures for breeding programs pull in multiple and sometimes contradictory directions. Shifting the balance toward social inclusion will require new and different incentive structures for breeding programs and seed companies alike, as well as heavy involvement by social scientists.

Furthermore, as this review indicates, addressing gender equality concerns in the context of agriculture and seed systems will require more than breeding. Attention to variety acceptability is warranted, but there are a plethora of gender issues surrounding seed availability, accessibility, and affordability, information access, and agency. The GENNOVATE Initiative provided pathways for more holistically evaluating women's needs, priorities, and constraints in accessing agricultural innovations, rather than only their preferences. In reference to maize seed, this will involve heightened attention to intrahousehold dynamics, women's agency in maize seed choice, and the impact of breeding-based solutions on women's empowerment—i.e., whether they reinforce women's restricted position in society or provide a meaningful step toward transforming gender relations and improving women's circumstances. A key challenge will be in pairing approaches that provide this depth of understanding with the large-scale quantitative studies of preferences and seed demand that breeders require.

Without a clearer path to operationalizing gender in maize breeding, it is likely to remain a talking point rather than a productive solution to women's seed access or gender equality. Breeding cycles and varietal replacement are moving faster than ever, but at least five to six years typically pass before new seeds reach farmers. Given this timeline, we urgently need to step back and seek a clearer understanding of how gender-intentional breeding fits among the range of potential entry points for improving women's use of new varieties and, more broadly, their empowerment. Otherwise, continued work on gender-intentional breeding may be misdirected. Clearly, however, to increase the relevance of gender and maize breeding work, researchers must immediately embrace innovative methods, better coordination, and critical questioning of underlying assumptions.

## Data Availability

There are no original data associated with this review.
